# Tetrameric structure of the restriction DNA glycosylase R.PabI in complex with nonspecific double-stranded DNA

**DOI:** 10.1038/srep35197

**Published:** 2016-10-12

**Authors:** Delong Wang, Ken-ichi Miyazono, Masaru Tanokura

**Affiliations:** 1 Department of Applied Biological Chemistry, Graduate School of Agricultural and Life Sciences, The University of Tokyo, Tokyo, Japan

## Abstract

R.PabI is a type II restriction enzyme that recognizes the 5′-GTAC-3′ sequence and belongs to the HALFPIPE superfamily. Although most restriction enzymes cleave phosphodiester bonds at specific sites by hydrolysis, R.PabI flips the guanine and adenine bases of the recognition sequence out of the DNA helix and hydrolyzes the *N*-glycosidic bond of the flipped adenine in a similar manner to DNA glycosylases. In this study, we determined the structure of R.PabI in complex with double-stranded DNA without the R.PabI recognition sequence by X-ray crystallography. The 1.9 Å resolution structure of the complex showed that R.PabI forms a tetrameric structure to sandwich the double-stranded DNA and the tetrameric structure is stabilized by four salt bridges. DNA binding and DNA glycosylase assays of the R.PabI mutants showed that the residues that form the salt bridges (R70 and D71) are essential for R.PabI to find the recognition sequence from the sea of nonspecific sequences. R.PabI is predicted to utilize the tetrameric structure to bind nonspecific double-stranded DNA weakly and slide along it to find the recognition sequence.

A type II restriction enzyme recognizes a specific double-stranded DNA (dsDNA) sequence and cleaves dsDNA at or near the sequence. Because most type II restriction enzymes cleave the phosphodiester bonds of dsDNA by hydrolysis in a sequence-dependent manner, they are also called restriction endonucleases^1^. Restriction enzymes are widely used in the field of biotechnology, such as in gene recombination, to cut dsDNA at specific sites for manipulation or analysis. Based on their structural features, type II restriction enzymes are categorized into several superfamilies: the PD-(D/E)XK superfamily^2^,^3^,^4^, the HNH superfamily^5^,^6^, the PLD superfamily^7^, the GIY-YIG superfamily^8^,^9^, and the HALFPIPE superfamily^10^,^11^. Although most restriction enzymes require Mg^2+^ ions for their dsDNA cleavage activities, enzymes within the PLD superfamily and the HALFPIPE superfamily cleave dsDNA without the addition of a divalent cation^7^,^12^. Restriction enzymes of the PLD superfamily cleave dsDNA using the phospholipase D-like active site, that which not require an Mg^2+^ ion to hydrolyze phosphodiester bonds^7^. On the other hand, restriction enzymes of the HALFPIPE superfamily do not cleave the phosphodiester bonds of dsDNA but cleave the *N*-glycosidic bonds of bases in a manner similar to DNA glycosylases^13^. R.PabI is a type II restriction enzyme from the hyperthermophilic archaea *Pyrococcus abyssi* and belongs to the HALFPIPE superfamily^10^,^12^,^13^. R.PabI homologs are only conserved among a hyperthermophilic archaea (*Staphylothermus hellenicus*), a thermophilic bacterium (*Caloramator australicus*), and some mesophilic bacteria such as *Helicobacter* and *Campylobacter* (Supplementary Fig. 1). R.PabI recognizes the 5′-GTAC-3′ sequence and cleaves the *N*-glycosidic bond of the adenine in the recognition sequence. Because the opposing apurinic/apyrimidinic (AP) sites generated by R.PabI are cleaved by β elimination and/or the endogenous AP endonucleases of host cells, R.PabI can cleave dsDNA at a specific site, similarly to restriction endonucleases. The structural analysis of R.PabI showed that R.PabI forms a dimer and possesses a characteristic highly curved β sheet, called the half-pipe structure, at the protomer-protomer interface^10^. R.PabI recognizes the 5′-GTAC-3′ sequence using the positively charged half-pipe structure. At the recognition sequence, R.PabI bends dsDNA by approximately 90^o^ and flips the adenine and guanine bases out of the DNA helix to recognize the sequence. The *N*-glycosidic bond of the flipped adenine is cleaved by R.PabI by hydrolysis, similarly to DNA glycosylases^13^.

In prokaryotic cells, restriction enzymes are utilized as protection from imported exogenous DNA. Due to the biological function of restriction enzymes, cells would suffer from lethal problems if restriction enzymes could not find their targets efficiently. In addition to restriction enzymes, other DNA binding proteins must also find their target sites efficiently to maintain cellular function. Therefore, the mechanisms by which DNA binding proteins find their target sites in the “sea” of nonspecific sequences within a short time are important for living cells, and these mechanisms of DNA binding proteins have been analyzed by several approaches including dynamic simulation^14^,^15^,^16^, structural analysis^2^,^4^,^17^ and single-molecule observation^18^,^19^,^20^. The common hypothesis generated by these experiments is that proteins could not feasibly find their target sites directly; instead, they first bind non-specifically and adjust their positions to find their target sites efficiently (facilitated diffusion)^21^,^22^,^23^,^24^. Several mechanisms of facilitated diffusion have been proposed: sliding, hopping, and intersegmental transfer. In sliding, proteins bind dsDNA non-specifically and diffuse along it linearly to seek their target sites^25^,^26^. In hopping, proteins dissociate from dsDNA, then diffuse in the solution and re-associate with nearby dsDNA^27^,^28^,^29^. In intersegmental transfer, proteins bind two dsDNA segments and transfer directly from one site to the other^30^,^31^.

Among restriction enzymes, the facilitated diffusion mechanisms of EcoRV and BamHI, which belong to the PD-(D/E)XK superfamily, have been well studied by several methods^2^,^4^,^17^,^29^,^32^. The structural analysis of the DNA free states, the specific dsDNA binding states, and the nonspecific dsDNA binding states of these enzymes showed that these proteins widen their DNA binding clefts when they bind nonspecific dsDNA, and their DNA binding clefts become narrow when they transform their structures into the specific binding states at their recognition sequences. These structural changes cause differences in the contacts between proteins and DNA; the contact surface areas of the specific dsDNA binding states are larger than the contact surface areas of the nonspecific dsDNA binding states. In addition, the nonspecific dsDNA binding states lack almost all of the base-specific interactions between proteins and DNA compared with the specific dsDNA binding states. The number of hydrogen bonds between proteins and phosphate groups of DNA are also decreased in the nonspecific dsDNA binding states compared with the specific dsDNA binding states. The wider DNA binding clefts, the smaller number of intermolecular hydrogen bonds, and the smaller contact surface area of the nonspecific dsDNA binding states of EcoRV and BamHI allow them to diffuse along dsDNA by lowering the activation energies for breaking and reforming DNA contacts^2^,^4^,^17^. Meanwhile, the mechanisms by which the HALFPIPE superfamily restriction enzymes find their recognition sequences have remained unclear because the structure of the nonspecific dsDNA binding state had not been determined.

In this study, we determined the crystal structure of R.PabI in complex with 20 base pairs of dsDNA, not containing the R.PabI recognition sequence, to uncover how R.PabI interacts with nonspecific dsDNA. The structure of the complex showed that R.PabI forms a tetrameric structure to sandwich dsDNA and the tetrameric structure is stabilized by four salt bridges. An electrophoretic mobility shift assay (EMSA) and DNA glycosylase assay of R.PabI mutants showed that the residues that form the salt bridges facilitates the DNA cleavage activity of R.PabI for dsDNA with an abundance of nonspecific sequences. The formation of the tetrameric structure is predicted to be important for R.PabI to find the recognition sequence efficiently in the sea of nonspecific sequences.

## Results

### Crystal structure of R.PabI in complex with nonspecific dsDNA

**Figure 1:**
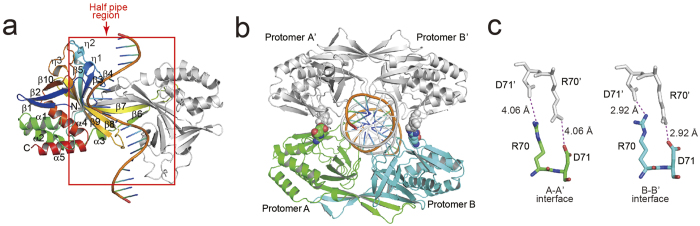
Overall structure of the R.PabI-nonspecific dsDNA complex. (**a**) Complex structure in the asymmetric unit. One R.PabI protomer is colored blue (in the N terminus) to red (in the C-terminus). The other R.PabI protomer is colored grey. The bound ssDNA is colored orange. Secondary structure assignments of protomer A are labelled on the model. The half-pipe region is indicated by a red box. (**b**) Tetrameric structure of the R.PabI-nonspecific dsDNA complex. R.PabI protomers A and B in one asymmetric unit are colored green and cyan, respectively. R.PabI protomers generated by a symmetry operation (protomers A’ and B’) are colored grey. The dsDNA between the two R.PabI dimers is colored orange (chain C) and grey (chain C’). R70 and D71, which form inter-dimer salt bridges, are shown by a sphere model. (**c**) Salt bridges between the two R.PabI dimers. Residues of protomers A and B are shown as green and cyan stick models, respectively. Residues of protomers A’ and B’ are shown as a grey stick model. Intermolecular salt bridges are shown as magenta dotted lines.

To determine the R.PabI-nonspecific dsDNA complex structure, we utilized the R32A E63A double mutant of R.PabI, which contains residues 8-226. Previous structural and mutational analyzes of R.PabI showed that the 1^st^~7^th^ residues of R.PabI are disordered in the structure^10^, and R32 and E63 participate in the stabilization and recognition of the flipped guanine base^13^. In addition, the R32A mutant lacks the sequence-specific DNA binding ability of R.PabI^10^. In this study, we determined the structure of R.PabI in complex with 20 bp of dsDNA at 1.9 Å resolution. The asymmetric unit of the crystal contains one R.PabI dimer (protomers A and B), one single-stranded DNA (ssDNA, chain C), and 162 water molecules (Fig. 1a, Table 1, and Supplementary Fig. 2a,b,c). Protomer A consists of five α helices, three 3_10_ (η) helices, and ten β strands. Among the β strands, strands β3, β4, β5, β6, β7, β8, and β9 form a half-pipe structure with the corresponding strands of protomer B. The structures of protomers A and B are nearly identical; the root mean square deviation (r.m.s.d.) between the two protomers is 1.7 Å for superposed 210 Cα atoms. The ssDNA is bound to the electropositive half-pipe region. The R.PabI-ssDNA complex observed in the asymmetric unit forms a tetrameric R.PabI structure with a symmetrically related complex generated by the crystallographic two-fold axis (R.PabI protomers A’ and B’ and ssDNA strand C’). The ssDNA in each asymmetric unit forms a dsDNA between the two R.PabI dimers (Fig. 1b).

In the tetrameric structure, the 20 bp dsDNA is sandwiched by the half-pipe regions of two R.PabI dimers. Although the contact surface area between the two protomers is small (the contact surface areas between the A-A’ and B-B’ interfaces are 201.1 Å^2^ and 102.8 Å^2^, respectively), the two R.PabI dimers are connected by four salt bridges between the protomers (Fig. 1c). At the A-A’ interface, R70 and D71 of protomer A form two salt bridges with D71′ and R70′ of protomer A’ at distances of 4.06 and 4.06 Å, respectively (the residues and bases of the symmetrically related molecules are indicated by a prime). At the B-B’ interface, R70 and D71 of protomer B form two salt bridges with D71′ and R70′ of protomer B’ at distances of 2.92 and 2.92 Å, respectively (Fig. 1c and Supplementary Fig. 2d,e).

### R.PabI-DNA interactions

**Figure 2:**
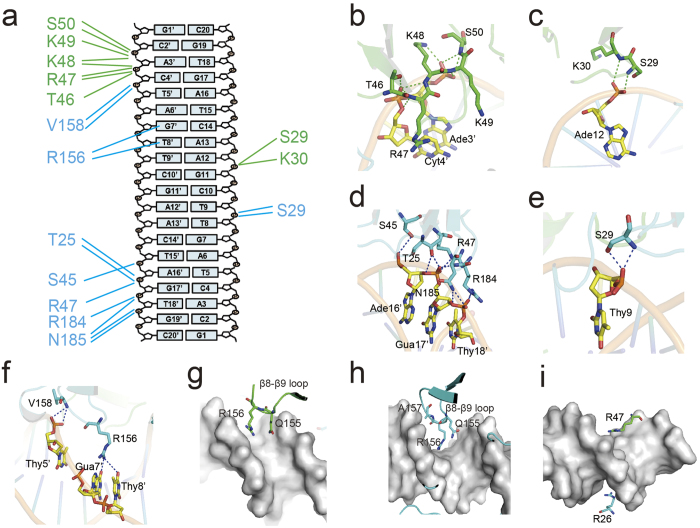
R.PabI-DNA interaction. (**a**) Intermolecular hydrogen bonds between one R.PabI dimer and dsDNA. Hydrogen bonds between protomer A and the dsDNA are shown as green lines. Hydrogen bonds between protomer B and the dsDNA are shown as cyan lines. Residues of protomers A and B are colored green and cyan, respectively. (**b**,**c**) Intermolecular hydrogen bonds between R.PabI protomer A and dsDNA. Hydrogen bonds are shown as green dotted lines. Residues of protomer A and DNA bases are colored green and yellow, respectively. (**d**–**f**) Intermolecular hydrogen bonds between R.PabI protomer B and dsDNA. Hydrogen bonds are shown as blue dotted lines. Residues of protomer B are colored cyan. (**g**–**i**) R.PabI residues located above the major or minor groove of the dsDNA. The dsDNA is shown as a grey surface.

**Figure 3:**
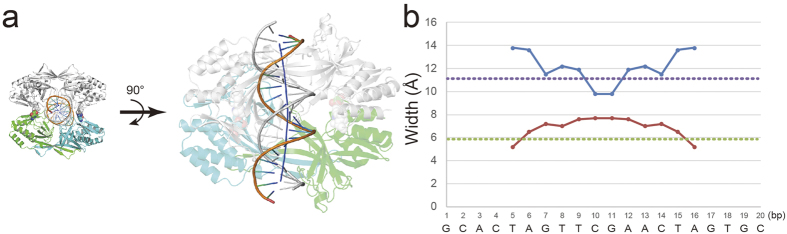
DNA structure in the R.PabI-nonspecific dsDNA complex. (**a**) The structure of dsDNA between the two R.PabI dimers. R.PabI and DNA are colored in the same way as Fig. 1. DNA axis is shown as a blue line. (**b**) Plots of major (blue line) and minor (red line) groove width as a function of DNA sequence. The major and minor groove widths of ideal B-form dsDNA are shown as purple and green dotted lines, respectively.

Similarly to EcoRV and BamHI^2^,^4^,^17^, the R.PabI dimer interacts with nonspecific dsDNA using small contact surface area and few hydrogen-bonds compared to the specific dsDNA. The interface area between the R.PabI dimer and the dsDNA in the nonspecific complex is 1156 Å^2^, which is approximately half of the interface area in the specific complex (2166 Å^2^)^13^. However, because R.PabI binds nonspecific dsDNA as a tetramer (Fig. 1b), the total R.PabI-dsDNA interface area of the nonspecific dsDNA binding state is approximately the same as in the specific dsDNA binding state. In the R.PabI-nonspecific dsDNA complex structure, the dsDNA is recognized by 24 hydrogen bonds between the DNA and each R.PabI dimer (Fig. 2a and Supplementary Table 1). The number of hydrogen bonds in the nonspecific complex is much lower than in the specific complex; the R.PabI dimer forms 69 hydrogen bonds with the specific dsDNA^13^. In protomer A, T46, R47, K48, K49, and S50 of the β4-η2 loop form eight hydrogen bonds with the phosphate groups of Ade3′ and Cyt4′ (Fig. 2b). S29 and K30 of the β2-β3 loop form two hydrogen bonds with the phosphate group of Ade12 (Fig. 2c). In protomer B, T25, S45, R47, R184, and N185 form eight hydrogen bonds with the phosphate groups of Ade16′, Gua17′, and Thy18′ (Fig. 2d). S29 and V158 form hydrogen bonds with the phosphate groups of Thy9 and Thy5′, respectively (Fig. 2e,f). Among these hydrogen bonding residues, T46, R47, V158, R184, and N185 also form hydrogen bonds with the phosphate groups of dsDNA in the R.PabI-specific dsDNA complex^13^. In addition to these sequence-nonspecific R.PabI-DNA hydrogen bonds, the side chain of R156 in protomer B is inserted into the major groove of dsDNA and forms two hydrogen bonds with the base groups of Gua7′ and Thy8′ (Fig. 2f). In the R.PabI-specific dsDNA complex structure, R156 plays an important role in recognizing the 5′-GTAC-3′ sequence, and its mutant shows decreased DNA cleavage activity^10^,^13^. The side chain of R156 in protomer B may be utilized to search for the R.PabI recognition sequence, especially the 5′-GT-3′ sequence. The structure of dsDNA between the two R.PabI dimers is slightly bent by ~20^o^ at its middle region due to these R.PabI-nonspecific dsDNA interactions (Fig. 3a). The minor groove of the dsDNA is expanded from a typical B-form dsDNA in the middle region (~8 Å) due to the dsDNA bending. On the other hand, the major groove of the dsDNA is narrowed from typical B-form dsDNA (~9 Å) in the middle region (Fig. 3b).

In addition to these hydrogen bonds, some residues are inserted into the major or minor grooves of dsDNA to recognize its shape or electrostatic potential, although these residues do not form any direct hydrogen bonds with the dsDNA. In the R.PabI-specific dsDNA complex structure, the R.PabI dimer bends dsDNA at the half-pipe region and unwinds the 5′-GTAC-3′ sequence for recognition using the β8-β9 loop^13^. In the R.PabI-nonspecific dsDNA complex structure, the β8-β9 loop of protomer A interacts with the phosphate backbone around the minor groove of Gua7-Cyt10, and the side chains of Q155 and R156, which are part of the sequence-specific interaction in the R.PabI-specific dsDNA complex structure, are inserted into the minor groove (Fig. 2g). The β8-β9 loop of protomer B interacts with the phosphate backbone around the major groove of Gua11-Ade16, and Q155, R156, and A157 are inserted into the major groove (Fig. 2h). In addition to R156 of protomer B that forms direct hydrogen bonds with bases, these residues are also predicted to be important in searching for the target sequence. Meanwhile, two arginine residues, R26 of protomer B and R47 of protomer A, are also inserted into the minor and the major groove of dsDNA, respectively (Fig. 2i). Some transcription factors insert arginine residues into minor grooves of dsDNA to recognize the sequence-specific shape and electrostatic potential of dsDNA to increase their sequence specificity^33^. Similarly, λ exonuclease was predicted to slide along dsDNA using an arginine residue inserted into the minor groove of dsDNA as a guide^34^. R26 of protomer B may also play an important role in the R.PabI-dsDNA interaction.

### Structure comparison between binding states

To analyze the structure modification of R.PabI upon the binding of dsDNA, the dimeric structures of R.PabI in the DNA-free state (PDB code: 2DVY)^10^, the nonspecific dsDNA binding state, and the specific dsDNA binding state (PDB code: 3WAZ)^13^ were superposed. When the structures of R.PabI in the DNA-free state and the nonspecific dsDNA binding state are compared, the structure of each protomer is observed to be slightly twisted in an anticlockwise direction in the nonspecific dsDNA binding state, and the structures of the β2-β3 loop of protomer B, which contains R26 as described above, and of the β4-η2 loop of each protomer are modified to form direct hydrogen bonds with the phosphate groups of the dsDNA (Supplementary Fig. 3a). On the other hand, the structures of the β8-β9 loops, which play an important role in unwinding dsDNA at the recognition sequence, are not modified by the binding of nonspecific dsDNA. The r.m.s.d. between the R.PabI dimers in the DNA free and the nonspecific dsDNA binding states is 2.2 Å for 424 superposed Cα atoms.

When the structures of R.PabI in the nonspecific dsDNA binding state and the specific dsDNA binding state are compared, the structure of each protomer is further twisted in an anticlockwise direction in the specific dsDNA binding state, and the structures of β2-β3, β4-η2, and β8-β9 loops of each protomer are modified to form hydrogen bonds with the deformed dsDNA (Supplementary Fig. 3b). In the specific dsDNA complex, P27, T28, and S29 of the β2-β3 loop protrude into the expanded minor groove of dsDNA to stabilize the unwound structure of dsDNA^13^. Although the position of R26 is slightly modified between the nonspecific dsDNA complex and the specific dsDNA complex, the side chain of R26 is inserted into the minor groove of dsDNA in each dsDNA binding state. The β4-η2 loop is utilized to form direct hydrogen bonds with the phosphate groups of dsDNA in each binding state. Because the dsDNA is bent by approximately 90° at the recognition sequence^13^, the positions of the phosphate groups differ between the nonspecific dsDNA complex and the specific dsDNA complex. The structure of β4-η2 loop is modified to optimize the interactions with the phosphate groups. The r.m.s.d. between the R.PabI dimers in the nonspecific dsDNA binding states and the specific dsDNA binding state is 2.4 Å for 427 superposed Cα atoms.

### Electrophoretic mobility shift assay

Although R.PabI binds the specific dsDNA sequence as a dimer^13^, the structure of the R.PabI-nonspecific dsDNA complex indicates that R.PabI binds nonspecific dsDNA as a tetramer and the tetrameric structure of R.PabI is stabilized by the four salt bridges. To analyze the DNA binding ability of R.PabI in solution, we performed the EMSA using the R.PabI active site mutant (Y68F), which possesses approximately the same base-specific DNA binding ability as the wild-type R.PabI but has reduced DNA glycosylase activity, as a control^13^.

**Figure 4:**
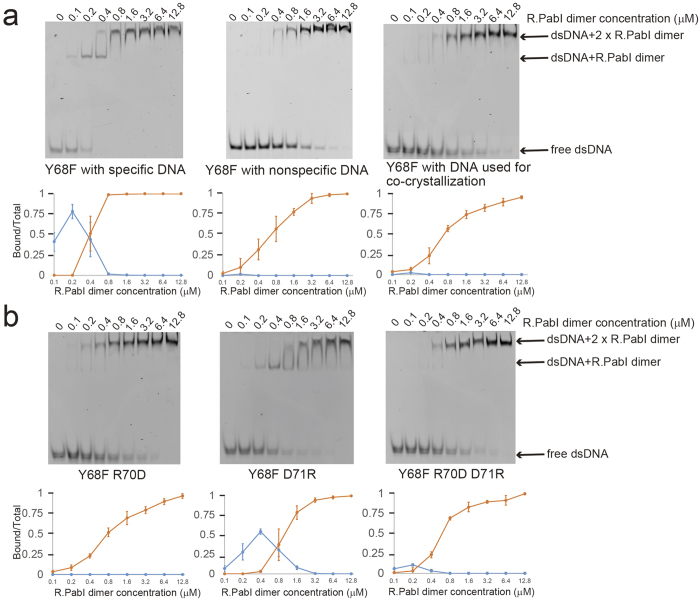
Electrophoretic mobility shift assay. (**a**) 0.1 μM of fluorescein-labelled dsDNA and each concentration of R.PabI were mixed and separated on a 12% polyacrylamide gel (cropped gel image). Quantifications show the ratio of DNA bound by the R.PabI dimer (blue) and the R.PabI tetramer (orange). Plotted values are mean ± SD (n = 3). (**b**) 0.1 μM of fluorescein-labelled dsDNA (the same sequence as the DNA used for co-crystallization) and each concentration of R.PabI were mixed and separated on a 12% polyacrylamide gel (cropped gel image).

First, we analyzed the sequence specific and the sequence nonspecific dsDNA binding ability of the R.PabI Y68F mutant (Fig. 4a and Supplementary Fig. 4). When we utilized the 20 bp dsDNA with the specific sequence as a probe, two shifted bands were observed: the shifted band observed at the low R.PabI concentration ranges and the super shifted band observed at the high R.PabI concentration ranges. Because R.PabI binds the recognition sequence as a dimer, the shifted band is predicted to present the R.PabI dimer-dsDNA complex. The super shifted band is predicted to present the R.PabI-DNA complex that is formed by sequence nonspecific R.PabI-DNA interactions due to the high concentration of R.PabI. In contrast, when we used the nonspecific dsDNA as a probe, the shifted band was very weak compared to the predominant super shifted band. Although the dsDNA that was utilized for the co-crystallization in this study contains a part of the R.PabI recognition sequences (5′-GT-3′, 5′-TA-3′ and 5′-AC-3′ steps), the DNA binding ability of R.PabI for the dsDNA used for the co-crystallization is approximately the same as that for the dsDNA not containing any 5′-GT-3′, 5′-TA-3′ and 5′-AC-3′ steps (Fig. 4a and Supplementary Fig. 4). This indicates that R.PabI does not recognize 5′-GT-3′, 5′-TA-3′ and 5′-AC-3′ steps in dsDNA except for 5′-GTAC-3′ (i.e. the dsDNA used for the co-crystallization is the nonspecific dsDNA for R.PabI).

Second, we analyzed the dsDNA binding ability of the R.PabI mutants to evaluate the importance of the salt bridge formations for the tetramerization of R.PabI. To prevent salt bridge formation, we produced the Y68F R70D and the Y68F D71R mutants. These mutations would prevent the tetramerization of R.PabI on nonspecific dsDNA due to the electrostatic repression of their side chains. In addition, the Y68F D71R mutant would also prevent the tetramerization of R.PabI on nonspecific dsDNA due to the steric hindrance of the long side chain of D71R. When the dsDNA binding ability of each mutant was analyzed by EMSA using the 20 bp DNA without the R.PabI recognition sequence as a probe, the Y68F and the Y68F R70D mutants showed approximately the same band shift pattern; only the super shifted bands were observed at all concentration ranges. In contrast, the Y68F D71R mutant showed a different band shift pattern; the shifted band was observed at the middle R.PabI concentration ranges (0.2–1.6 μM of the R.PabI dimer, Fig. 4b and Supplementary Fig. 4) in addition to the super shifted band. Because the structural analysis of R.PabI-nonspecific dsDNA complex showed that R.PabI dimer binds 20 bp dsDNA as a tetramer, the super shifted bands observed in the Y68F and the Y68F R70D mutants were predicted to represent the complex of two R.PabI dimers and a labelled dsDNA, and the shifted bands observed in the result of the Y68F D71R binding assay were predicted to represent the complex of one R.PabI dimer and a labelled dsDNA. These results indicate that the tetramerization of R.PabI is inhibited by the D71R mutation, although the R70D mutation showed no effect on the nonspecific dsDNA binding affinity, and that R.PabI prefers to bind nonspecific 20 bp dsDNA not as a dimer but as a tetramer. The gel filtration analysis also showed that R.PabI forms a tetramer with nonspecific dsDNA and the tetramerization is prevented by the D71R mutation (Supplementary Fig. 5). Because the side chain length of aspartic acid is shorter than for arginine, the effect of electrostatic repression between R70D and D71 would not be enough to block the binding of the second R.PabI dimer on nonspecific dsDNA. The tetramerization of R.PabI on nonspecific dsDNA is predicted to be mainly accelerated by the deformation of dsDNA structure caused by a binding of one R.PabI dimer and the resulting tetrameric structure of R.PabI is stabilized by the four salt bridges. The deformation of dsDNA structure is an important factor for cooperative bindings of multiple DNA binding proteins^35^,^36^. We also analyzed the DNA binding ability of the Y68F R70D D71R triple mutant. Because the R70D D71R mutation would form two salt bridges at the interface region, similarly to the wild type enzyme, the inhibition of the tetramerization of R.PabI by the D71R mutation would be recovered by the R70D D71R mutation. The Y68F R70D D71R mutant showed similar nonspecific dsDNA binding ability to the control Y68F mutant, as expected (Fig. 4b and Supplementary Fig. 4).

### DNA glycosylase assays using dsDNA with different lengths

**Figure 5:**
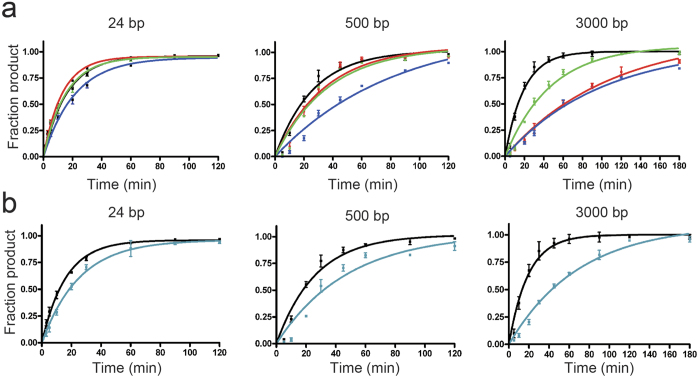
DNA glycosylase assay using various lengths of dsDNA. (**a**) DNA glycosylase assays of the Y68F (black), Y68F R70D (red), Y68F D71R (blue), and Y68F R70D D71R (green) mutants using 24 bp, 500 bp, and 3000 bp dsDNA as substrates. Plotted values are mean ± SD (n = 3). (**b**) DNA glycosylase assays of the Y68F (black) and R26A Y68F (cyan) mutants using 24 bp, 500 bp, and 3000 bp dsDNA as substrates. Plotted values are mean ± SD (n = 3).

To evaluate the importance of the tetramerization of R.PabI on nonspecific dsDNA for its enzymatic activity, we analyzed the DNA glycosylase activities of the Y68F, Y68F R70D, Y68F D71R, and Y68F R70D D71R mutants using dsDNA with different lengths (Supplementary Fig. 6a). The results of the DNA glycosylase assay of the Y68F mutant showed that the activity of the Y68F mutant for the 3000 bp dsDNA was higher than that for the 500 bp dsDNA (Table 2), although the 3000 bp dsDNA contains more nonspecific dsDNA per R.PabI recognition sequence than the 500 bp dsDNA. Similar results were also observed in other restriction enzymes that utilized the facilitated diffusion mechanisms to find their targets; DNA cleavage activities of these enzymes are increased when substrate dsDNA sequences contain more nonspecific sequences^37^. If R.PabI utilizes the tetrameric structure observed in the R.PabI-nonspecific dsDNA complex structure to facilitate diffusion, the DNA glycosylase activities of the mutants that prevent tetramerization on nonspecific dsDNA (the Y68F R70D and Y68F D71R mutants) would be reduced with increasing lengths of nonspecific dsDNA per R.PabI recognition sequence and the reduced activity would be recovered by the R70D D71R mutation. When the 24 bp dsDNA was utilized as a substrate, the Y68F R70D, Y68F D71R, and Y68F R70D D71R mutants showed 127%, 74% and 105% relative activities compared to the control Y68F mutant (Table 2, Fig. 5a and Supplementary Fig. 6). When the 500 bp and 3000 bp dsDNA were utilized as substrates, the Y68F R70D mutant showed 76% and 19% relative activities compared to the Y68F mutant, respectively. The activity of the Y68F D71R mutant was also decreased to 34% and 19% of the Y68F mutant when using the 500 bp and 3000 bp dsDNA as substrates, respectively. These data showed that the mutations of R70D and D71R result in decreased DNA glycosylase activities for longer dsDNA substrates. Meanwhile, the decreased activities for longer dsDNA substrates were recovered by the double mutation of R70D D71R as expected; the activities of the Y68F R70D D71R mutant for the 500 bp and 3000 bp dsDNA were 71% and 40% of the activity of the Y68F mutant (Table 2, Fig. 5a, and Supplementary Fig. 6).

We also analyzed the DNA glycosylase activities of the Y68F R26A mutants for dsDNA with different lengths. In the structure of the nonspecific dsDNA complex, Arg26 is located above the minor groove of dsDNA (Fig. 2g) and is predicted to play an important role in the R.PabI-dsDNA interaction. When the 24 bp dsDNA was utilized as a substrate, the Y68F R26A mutant showed 65% relative DNA glycosylase activity compared to the control Y68F mutant. The Y68F R26A mutant showed 55% and 27% relative activities compared to the Y68F mutant when using the 500 bp and 3000 bp dsDNA as substrates, respectively (Table 2, Fig. 5b and Supplementary Fig. 6). R26 is also predicted to play an important role in cleaving long dsDNA substrates efficiently.

## Discussion

In this study, we have determined the crystal structure of the R.PabI-nonspecific dsDNA complex at 1.9 Å resolution. In the complex structure, R.PabI forms a tetrameric structure on nonspecific dsDNA using four salt bridges, although it binds dsDNA as a dimer at the recognition sequence 13. The EMSA and DNA glycosylase activity assays using the R.PabI mutants showed that the residues that stabilize the tetrameric structure by the salt bridges are important for finding the recognition sequence in the sea of nonspecific dsDNA sequences. Restriction endonucleases such as EcoRV and BamHI bind nonspecific dsDNA weakly using their widened dsDNA binding cleft to search for their recognition sequences; each restriction endonuclease modifies the structure at their recognition sequences to form base-specific DNA interactions and to hydrolyze phosphodiester bonds at specific sites^2^,^4^,^17^. Our results in this report indicates that the restriction DNA glycosylase R.PabI also utilizes the nonspecific dsDNA binding state to facilitate diffusion similarly to EcoRV and BamHI, but the tetramerization on nonspecific dsDNA is a unique feature of R.PabI. EcoRV and BamHI, as well as most other restriction enzymes except for NgoMIV, which forms a tetramer at the product binding state^38^, bind dsDNA as dimers in both the specific and the nonspecific dsDNA binding states.

At the nonspecific dsDNA binding state, two R.PabI dimers bind the same region of nonspecific dsDNA to form the tetrameric structure. The tetrameric structure is stabilized by four salt bridges using R70 and D71 of each protomer. The crystal structure of the R.PabI-nonspecific dsDNA complex shows that the structure of dsDNA between the two R.PabI dimers is deformed by the binding of R.PabI, even in the nonspecific dsDNA binding state (Fig. 3). Although R.PabI may also bind nonspecific dsDNA as a dimer, similarly to EcoRV and BamHI, the deformed structure of nonspecific dsDNA by the binding of one R.PabI dimer would facilitate the binding of the other R.PabI dimer at the same site because the two R.PabI dimers bind the same deformed nonspecific dsDNA site symmetrically, and the resulting tetrameric structure is stabilized by salt bridges (Fig. 1b). Actually, the results of EMSA showed that R.PabI binds nonspecific dsDNA as a tetramer, except for the Y68F D71R mutant, which would interfere with the tetramerization of R.PabI by both electrostatic repulsion and steric hindrance (Fig. 4). Sequence-specific DNA binding proteins search for their target sequences using a sliding mechanism that facilitates 1D diffusion along nonspecific dsDNA^25^,^26^ and/or a hopping mechanism that facilitates 3D diffusion^27^,^28^,^29^. Because the tetramerization of R.PabI stabilizes the R.PabI-nonspecific dsDNA complex using the toroidal structure, the tetrameric structure of R.PabI might facilitate not the hopping efficiency but the sliding efficiency for seeking the recognition sequence.

Although the dsDNA is bent approximately 90^o^ at the recognition sequence^13^, the structure of dsDNA is not so modified at the nonspecific dsDNA binding state; the dsDNA structure of the nonspecific site is not unwound by the binding of R.PabI, and the structures of the β8-β9 loops, which unwind dsDNA, are rarely modified compared to the DNA-free structure of R.PabI. R.PabI would deform the structure of dsDNA only when it arrives at the recognition sequence. However, the structure of dsDNA is slightly modified by the binding of R.PabI to widen its minor groove even in the nonspecific dsDNA binding state (Fig. 3). Because the DNA bending at the recognition sequence occurs after the insertion of the β8-β9 loop into the minor groove, the widened minor groove would facilitate the deformation of dsDNA at the recognition sequence. The β8-β9 loop existing on the minor groove of dsDNA observed in the complex structure (protomer A) may be inserted into the minor groove of dsDNA at the recognition sequence to unwind the dsDNA structure.

In this study, we have shown that the salt bridges formed between R70 and D71 are important for the nonspecific dsDNA dependent tetramerization of R.PabI. However, these two residues are not conserved among the R.PabI homologs except the one from hyperthermophilic archaea *Staphylothermus hellenicus* (Supplementary Fig. 1). Some thermostable proteins form multimers to increase their thermal stabilities^39^,^40^,^41^. Because R.PabI and its homolog from *S. hellenicus* function at high temperature, these proteins may utilize tetrameric structures to increase the stability of the protein-nonspecific dsDNA complex and thus facilitate sliding along dsDNA to search for their recognition sequences efficiently.

## Materials and Methods

### Protein expression and purification

The gene fragment of the R.PabI R32A E63A mutant, which contains residues 8-226, was amplified by PCR and cloned into the NdeI-BamHI site of pET26b plasmid (Novagen). The constructed plasmid was transformed into *E. coli* Rosetta(DE3)pLysS (Novagen) for protein expression. The recombinant *E. coli* cells that overexpressed R.PabI R32A E63A were resuspended in 25 mM MES (pH 6.0) and 50 mM MgCl_2_ and were lysed by sonication. After centrifugation at 40,000 × g for 30 min, the supernatant was treated with Cryonase Cold-active Nuclease (TAKARA) to remove contaminant nucleic acids from *E. coli*. The solution was heated at 80 °C for 30 min to denature heat-labile *E. coli* proteins and then centrifuged at 40,000 × g for 30 min. The supernatant was applied onto a Toyopearl AF-Heparin-650M (TOSOH) column. The bound R.PabI was eluted with 10 mM MES (pH 6.0) and 1 M NaCl. The eluted protein was further purified using a MonoS HR 10/10 (GE Healthcare) column pre-equilibrated with 10 mM MES (pH 6.0) and was eluted using a linear gradient of 0-1 M NaCl. After the purification, the protein buffer was exchanged with 10 mM MES (pH 6.0) and 100 mM NaCl. The protein solutions were concentrated to ~100 μM (the dimer concentration) and were stored at −80 °C until use.

The R.PabI Y68F mutant, the R.PabI Y68F R70D mutant, the R.PabI Y68F D71R mutant, the R.PabI Y68F R70D D71R mutant, and the R.PabI Y68F R26A mutant, containing residues 8-226, were prepared by modifying the pET26b-R.PabI R32A E63A plasmid using the PrimeSTAR Mutagenesis Basal kit (TAKARA). The modified plasmids were transformed into *E. coli* Rosetta(DE3)pLysS for protein expression. The expression and purification of these mutants were performed using the same method as for R.PabI R32A E63A. The purified protein solutions were stored at −80 °C until use.

### Double-stranded DNA Preparation

The oligonucleotide purification cartridge (OPC)-purified oligonucleotides were purchased from Eurofins Genomics and were dissolved in the annealing solution containing 2.5 mM MES (pH 6.0), 20 mM NaCl, and 2.5 mM MgCl_2_ to be 25 μM. The ssDNA samples were annealed by incubating at 368 K and slow cooling to 277 K.

### Crystallization and structure determination

For the co-crystallization of the R.PabI R32A E63A-nonspecific dsDNA complex, 20 bp blunt-ended dsDNA (5′-GCACTAGTTCGAACTAGTGC-3′, Supplementary Fig. 2a) was mixed with the R.PabI R32A E63A dimer in a molar ratio of 1:2 in 10 mM MES (pH 6.0) and 100 mM NaCl. The mixture was concentrated to 156 μM (the concentration of the R.PabI dimer). Crystallization experiments with the R.PabI R32A E63A-nonspecific dsDNA complex were performed using the sitting-drop vapour-diffusion method at 20 °C. The crystals of the R.PabI R32A E63A-nonspecific dsDNA complex were obtained using a reservoir solution of 0.2 M calcium acetate, 0.1 M imidazole (pH 8.0), and 10% PEG8000.

X-ray diffraction data were collected on the AR-NE3A beamline at the Photon Factory (Tsukuba, Japan) under cryogenic conditions (95 K). For cryoprotection, the crystal of the R.PabI R32A E63A-nonspecific dsDNA complex was soaked in a reservoir solution supplemented with 30% glycerol for a few seconds. The crystal of the R.PabI R32A E63A-nonspecific dsDNA complex diffracted X-rays to 1.9 Å resolution. The X-ray diffraction data were indexed and integrated using the programme XDS^42^ and scaled using SCALA in the CCP4 suite^43^. The crystal of the R.PabI R32A E63A-nonspecific dsDNA complex belongs to the space group *C*222_1_ with unit cell parameters of *a* = 72.89, *b* = 261.7, and *c* = 65.08 Å. The initial model was determined by the molecular replacement method using the programme MOLREP^44^ with the coordinates of the DNA-free R.PabI structure (PDB code: 2DVY)^10^. The initial model was refined and rebuilt using the programmes Phenix.refine^45^ and Coot^46^. The final model of the R.PabI R32A E63A-nonspecific dsDNA complex was refined to 1.9 Å resolution with *R* and *R*_free_ values of 20.62% and 24.81%, respectively. The geometry of the final model was evaluated using the programme MolProbity^47^. In the Ramachandran plot, 97.2% of the residues were included in the favoured region, and the remaining residues were in the allowed region. The data collection and refinement statistics are summarized in Table 1. Although the *R*_sym_ is high for the highest resolution shell, the mean *I*/sig*I* is not smaller than 2, indicating that the resolution limit of the structure determination is reasonable. The structure of the R.PabI R32A E63A-nonspecific dsDNA complex was depicted and superposition of two protein structures performed using the programme Pymol ( http://www.pymol.org). Intermolecular interactions between the R.PabI R32A E63A mutant and the dsDNA were analyzed using the programme PISA^48^. The composite omit map was generated using the CCP4 suite^43^. The width of the dsDNA groove was calculated using Curves+^49^.

### Electrophoretic mobility shift assay

Three 5′-fluorescein-labelled 20 bp dsDNAs (5′-GCACTAGTTCGAACTAGTGC-3′ (same sequence as the DNA used for co-crystallization), 5′-GCATCGATTCGAATCGATGC-3′ (nonspecific), and 5′-GCATAGCTGTACAGCTATGC-3′ (specific)) were used as probes. 0.1 μM of each DNA probe and the R.PabI dimer (0.1, 0.2, 0.4, 0.8, 1.6, 3.2, 6.4, and 12.8 μM) were mixed in 10 mM MES (pH 6.0), 300 mM NaCl, and 50 μM single-stranded poly-dT (40 nt) and incubated for 30 min at 4 °C. To detect the R.PabI-dsDNA interactions by EMSA, we added an abundance of ssDNA as a competitor. Bound and unbound dsDNA were separated through a 12% polyacrylamide gel in 0.5 × TBE at 4 °C, and fluorescence was measured using an LAS4000 Mini system (Fujifilm, Tokyo, Japan).

### Oligomeric state analysis by gel filtration chromatography

5 μM of the R.PabI dimers and 2.5 μM of the nonspecific dsDNA (5′-GCACTAGTTCGAACTAGTGC-3′) were mixed in 10 mM MES (pH 6.0) and 300 mM NaCl. 5 μM of the R.PabI dimers (Y68F, R32A E63A, and Y68F R70D), 2.5 μM of the nonspecific dsDNA, and the R.PabI-nonspecific dsDNA mixtures were loaded onto a Superdex 200 HR 10/30 (GE Healthcare) column and were eluted with buffer containing 10 mM MES (pH 6.0) and 300 mM NaCl. 5 μM of the R.PabI dimers (Y68F D71R and Y68F R70D D71R) were loaded onto a Superdex 200 HR 10/30 (GE Healthcare) column and were eluted with buffer containing 10 mM MES (pH 6.0) and 600 mM NaCl to avoid nonspecific binding to the Superdex 200 column. To estimate the oligomeric state of R.PabI, the following standard proteins were used: ferritin (*Mr *= 440,000), aldolase (*Mr *= 158,000), conalbumin (*Mr *= 75,000), ovalbumin (*Mr *= 44,000), and ribonuclease A (*Mr *= 13,700).

### DNA glycosylase assay

DNA glycosylase activity assays of the R.PabI mutants were performed using 24 bp, 500 bp, and 3000 bp dsDNAs possessing only one R.PabI recognition sequence at their center regions. The 5′-fluorescein-labelled 24 bp dsDNA (5′-fluorecesin-GGATGCATGAGTACGAGGACCATC-3′, Supplementary Fig. 6a) was purchased from Eurofins. The 500 bp and 3000 bp dsDNA were amplified by PCR using the modified pET26b plasmid, which has only one 5′-GTAC-3′ site in the *lacI*-coding region^13^, as a template and purified using the QIAquick PCR Purification Kit (QIAGEN). Then, 0.2 μM of the 24 bp dsDNA was mixed with 0.4 μM of the R.PabI dimers in a reaction buffer containing 0.1 M phosphate buffer (pH 6.5), while 5.9 nM of the 500 bp or 3000 bp dsDNA was mixed with 80 nM of the R.PabI dimers in reaction buffer containing 0.1 M phosphate buffer (pH 6.5). The reaction solution was incubated at 45 °C for 1, 3, 5, 10, 20, 30, 60, 90, and 120 min (for the 24 bp dsDNA substrate), 5, 10, 20, 30, 45, 60, 90, and 120 min (for the 500 bp DNA substrate), or 5, 10, 20, 30, 45, 60, 90, 120, and 180 min (for the 3000 bp DNA substrate). After the enzymatic reactions, the reaction solutions were supplemented with 0.1 M NaOH to stop the enzymatic reaction. The solutions were then heated at 70 °C for 10 min to cleave the products at the 5′ and 3′ side of the AP sites generated by R.PabI and neutralized by the addition of an equal concentration of HCl. The reaction solutions using 24 bp dsDNA as a substrate were separated on a denaturing 18% polyacrylamide gel in 0.5 × TBE and 7 M urea. The reaction solutions using 500 bp or 3000 bp dsDNA as a substrate were separated on a 1% agarose gel and stained using GelGreen (Biotium). The fluorescence was measured using an LAS4000 Mini system. The enzymatic rate constant *k* was obtained from a single-exponential fit to the data from three independent measurements: *f*_p _= *f*_p_max × (1 − e^−*kt*^), in which *f*_p_ is the fraction of product, *f*_p_max is the maximum value of *f*_p_, and *t* is the time of the reaction.

## Additional Information

**Accession code**: Coordinates and structural factors are deposited in the PDB under the accession codes 5IFF.

**How to cite this article**: Wang, D. *et al*. Tetrameric structure of the restriction DNA glycosylase R.PabI in complex with nonspecific double-stranded DNA. *Sci. Rep.* **6**, 35197; doi: 10.1038/srep35197 (2016).

## Supplementary Material

Supplementary Information

## Figures and Tables

**Table 1:** Summary of data collection and refinement statistics of the R.PabI-nonspecific dsDNA complex.

Data collection	
X-ray source	Photon Factory AR-NE3A
Detector	ADSC Quantum270
Wavelength (Å)	1.0000
Space group	C222_1_
Unit cell parameters	
*a, b, c* (Å)	72.89, 261.7, 65.08
α, β, γ (°)	90, 90, 90
Resolution (Å)	43.6–1.90 (1.94–1.90)*
Wilson B-factor (Å^2^)	41.5
Total number of reflections	685056 (27971)
Unique reflections	49388 (3110)
*R*_sym_ (%)	5.1 (111.1)
Mean *I/σI*	28.5 (2.0)
Completeness (%)	99.6 (99.2)
Multiplicity	13.9 (9.0)
**Refinement**	
Resolution (Å)	43.6–1.90 (1.94–1.90)
No. of reflections in working/test set	49246/2444
*R/R*_free_ (%)	20.62/24.81 (34.07/34.00)
No. atoms	
Protein/DNA/water	3475/407/162
*B*-factors (Å^2^)	
Chain A (protomer A)	48.96
Chain B (protomer B)	74.82
DNA (chain C)	57.20
water	53.70
r.m.s.d.	
Bond angle (°)	1.30
Bond length (Å)	0.013
Rotamer outliers (%)	0.80
C-beta outliers	0
Clash score	3.91

^*^The numbers in parentheses represent data for the highest-resolution shells.

**Table 2:** DNA glycosylase activities of the mutants.

Mutants	Substrate dsDNA	*k* (min^−1^)*	Relative activity (%)
Y68F (control)	24 bp	0.062 ± 0.003	100
	500 bp	0.038 ± 0.007	100
	3000 bp	0.052 ± 0.006	100
Y68F R70D	24 bp	0.079 ± 0.004	127
	500 bp	0.029 ± 0.008	76
	3000 bp	0.010 ± 0.001	19
Y68F D71R	24 bp	0.046 ± 0.003	74
	500 bp	0.013 ± 0.004	34
	3000 bp	0.010 ± 0.002	19
Y68F R70D D71R	24 bp	0.065 ± 0.005	105
	500 bp	0.027 ± 0.007	71
	3000 bp	0.021 ± 0.003	40
Y68F R26A	24 bp	0.040 ± 0.001	65
	500 bp	0.021 ± 0.006	55
	3000 bp	0.014 ± 0.002	27

^*^The enzymatic rate constants and their standard errors were obtained from a single-exponential fit to the data from three independent measurements.
