# Supplementary material for: Tetrameric structure of the restriction DNA glycosylase R.PabI in complex with nonspecific double-stranded DNA
Source: Sci Rep. 2016 Oct 12;6:35197. doi: 10.1038/srep35197 (PMC5059719; doi:10.1038/srep35197)
Supplement: Supplementary Information [file srep35197-s1.pdf]

Tetrameric structure of the restriction DNA glycosylase R.PabI in complex with nonspecific double-stranded DNA

Delong Wang, Ken-ichi Miyazono, and Masaru Tanokura*

Department of Applied Biological Chemistry, Graduate School of Agricultural and Life Sciences, The University of Tokyo, Tokyo, Japan

*To whom correspondence should be addressed. Tel: +81-3-5841-5165; Fax: +81-3-5841-8023; Email: amtanok@mail.ecc.u-tokyo.ac.jp

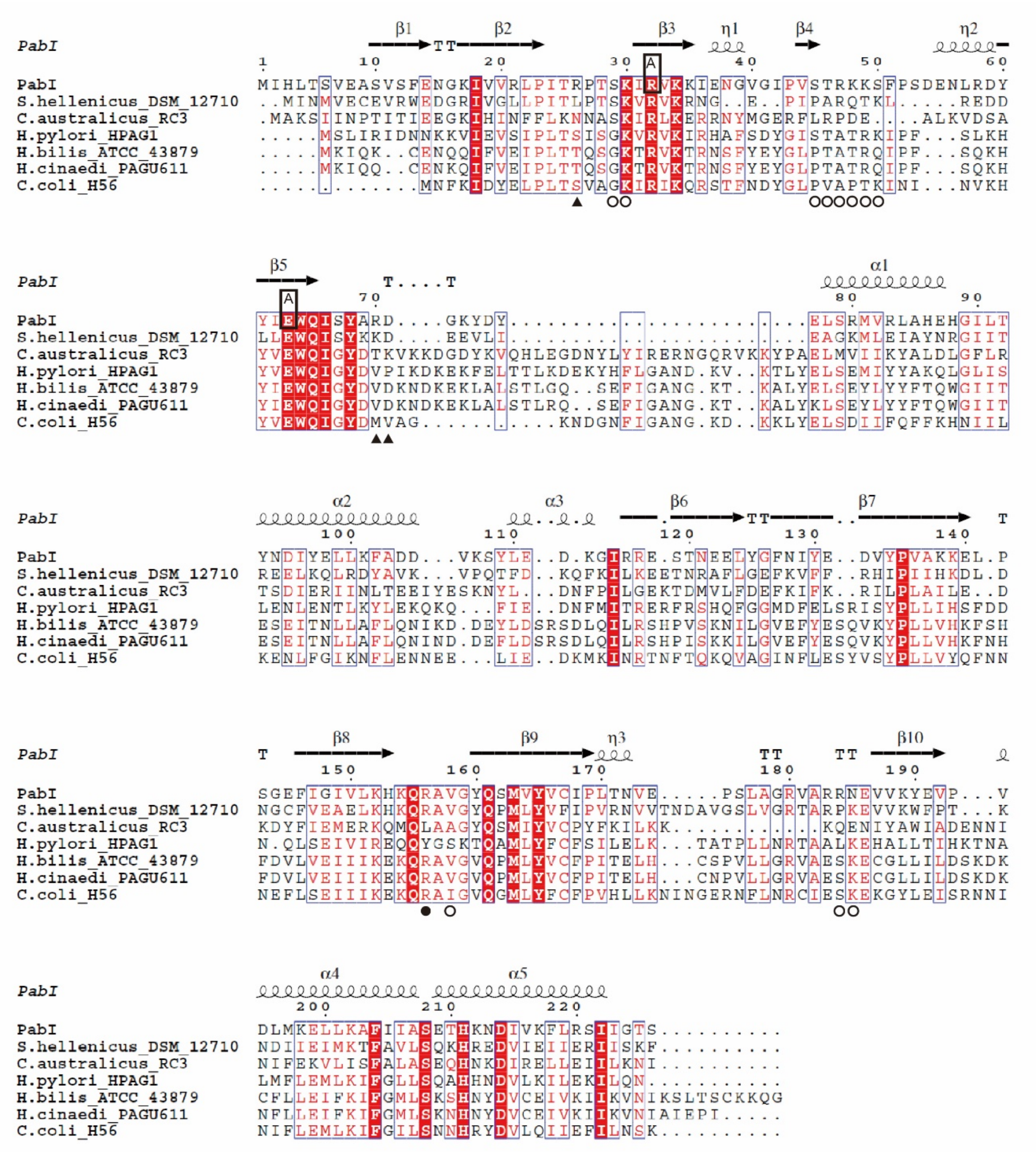

Supplementary Figure 1. Amino acid sequence alignment of R.PabI and its homologues. Invariant residues are highlighted with red boxes, and conserved residues are shown in red text. The secondary structure of R.PabI in the R.PabI-nonspecific dsDNA complex is indicated by helices (α and η (3_{10})-helices), arrows (β-strand), and TT (β-turn). Residues that were mutated to alanine for the structure determination are indicated by black boxes. Residues that recognize the DNA bases and the sugar-phosphate backbones are marked with black and open circles, respectively. Residues analysed by mutagenesis are marked with black triangles.

a

```
    1  2  3  4  5  6  7  8  9  10 11 12 13 14 15 16 17 18 19 20
5'- G  C  A  C  T  A  G  T  T  C  G  A  A  C  T  A  G  T  G  C -3'
3'- C  G  T  G  A  T  C  A  A  G  C  T  T  G  A  T  C  A  C  G -5'
    20'19'18'17'16'15'14'13'12'11'10' 9' 8' 7' 6' 5' 4' 3' 2' 1'
```

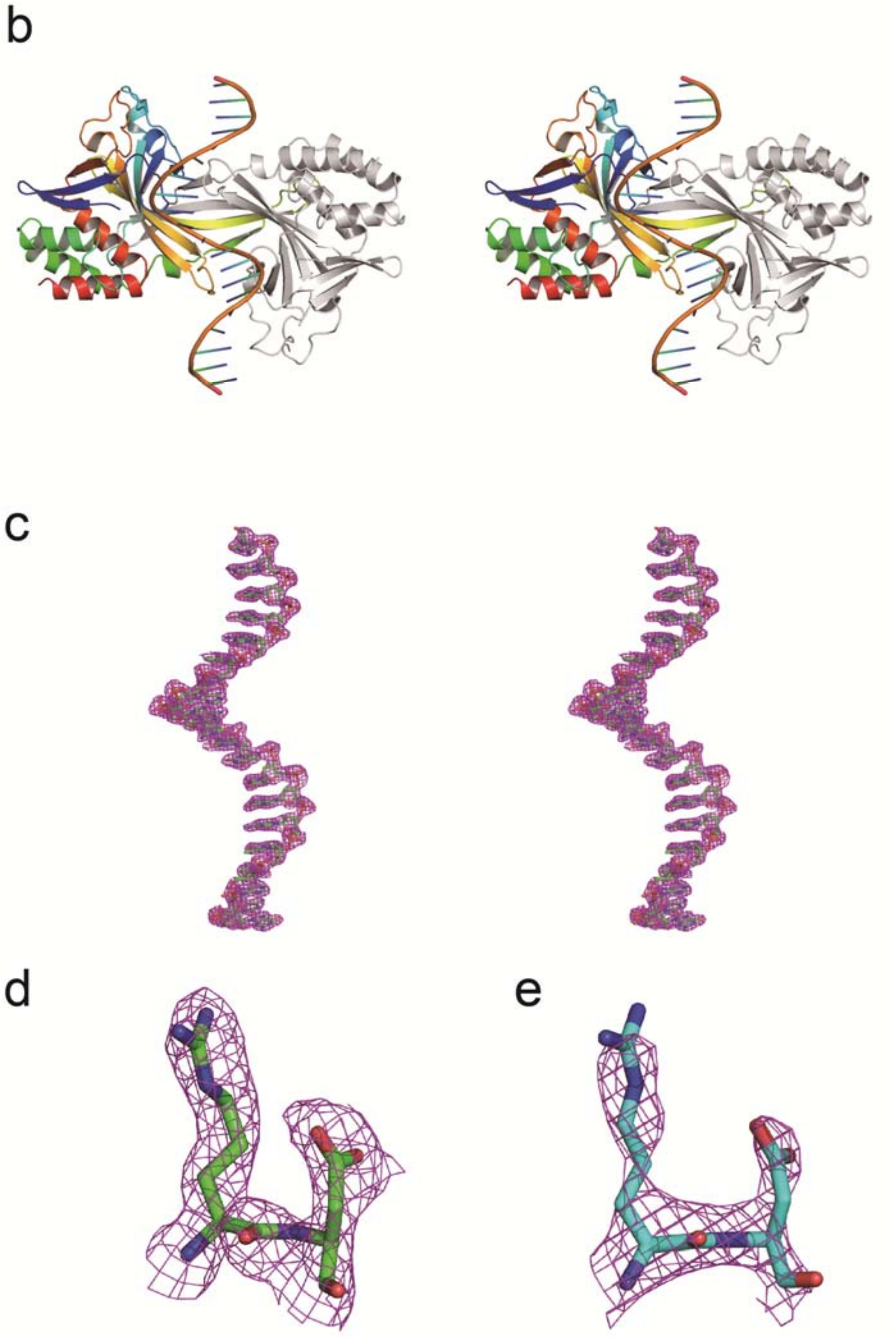

Supplementary Figure 2. Structure determination of the R.PabI-nonspecific dsDNA complex. (**a**) Sequence of a dsDNA used for the structure determination of the R.PabI-nonspecific dsDNA complex. (**b**) Wall-eyed stereo image of the R.PabI-nonspecific dsDNA complex in one asymmetric unit. (**c**) Wall-eyed stereo image of the bound ssDNA and its composite omit map (1.7 σ, magenta). The structure of DNA is shown as a stick model. (**d**) Composite omit map (1.7 σ, magenta) of Arg70 and Asp71 of protomer A. (**e**) Composite omit map (1.7 σ, magenta) of Arg70 and Asp71 of protomer B.

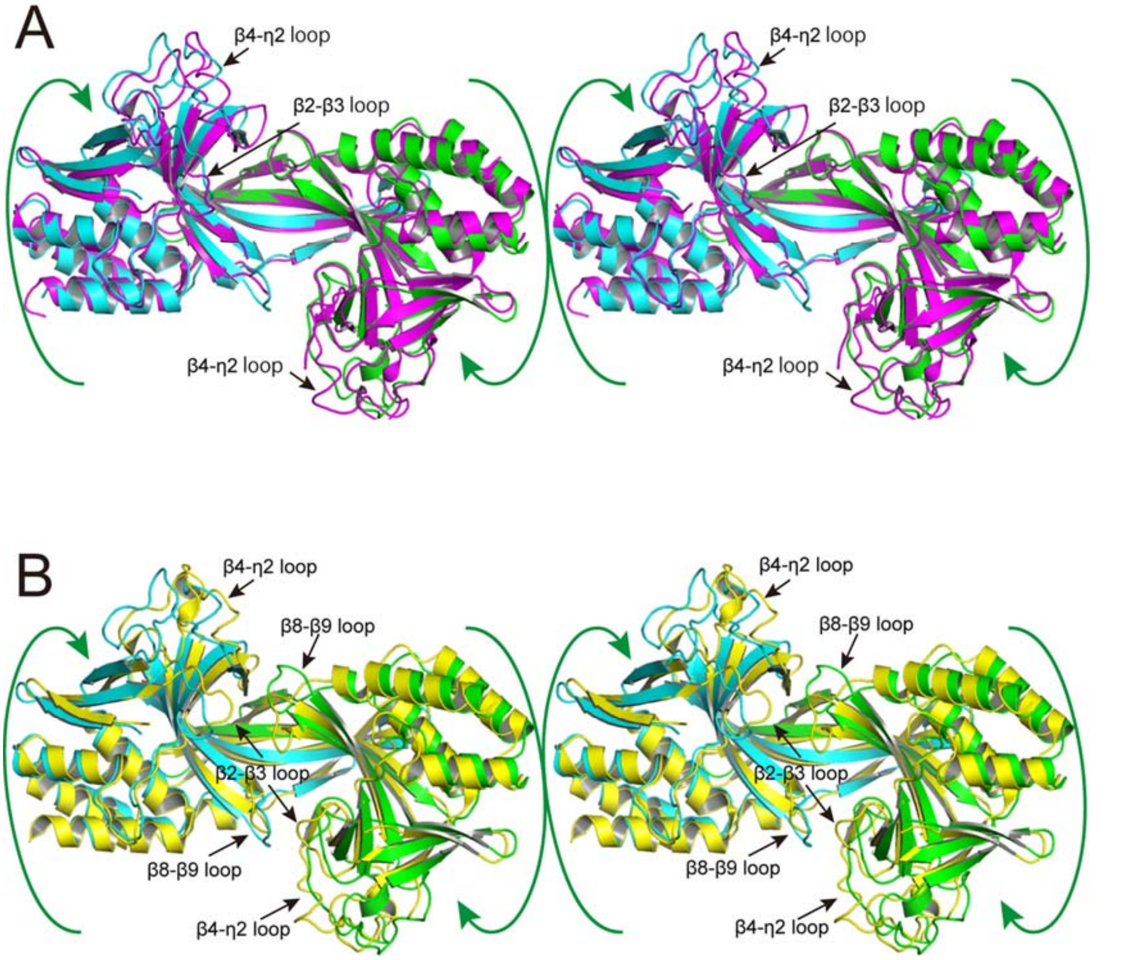

Supplementary Figure 3. Structure comparison of R.PabI in the DNA-free state, the nonspecific dsDNA binding state, and the specific dsDNA binding state. (**a**) Wall-eyed stereo image of the superposition of the DNA-free state (magenta) and the nonspecific DNA binding state (green and cyan) of the R.PabI dimers. (**b**) Wall-eyed stereo image of the superposition of the nonspecific DNA binding state (green and cyan) and the specific DNA binding state (yellow) of the R.PabI dimers.

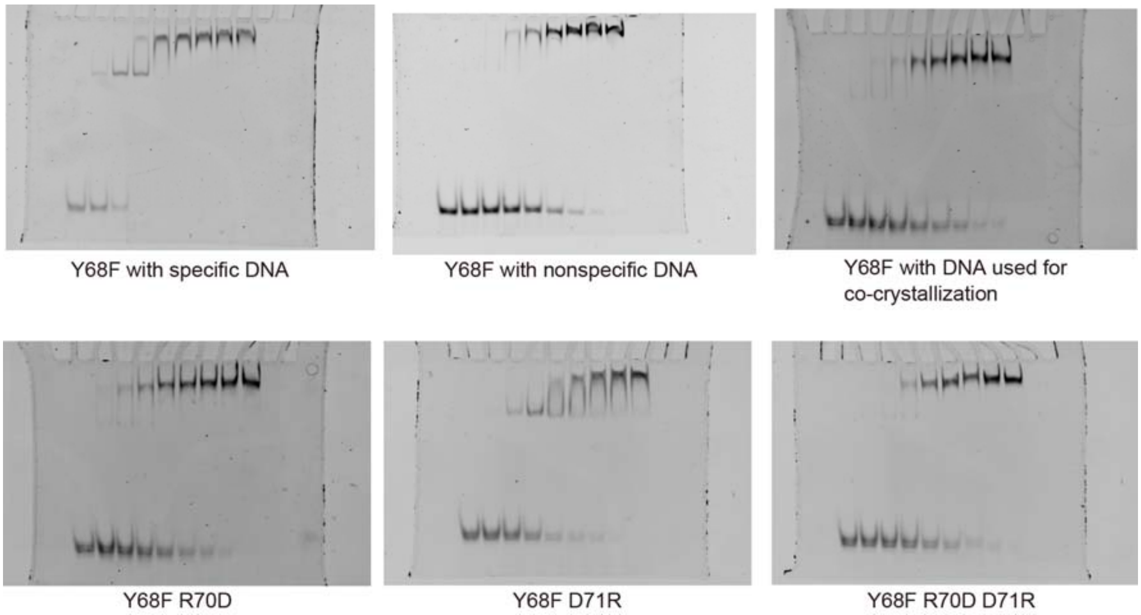

Supplementary Figure 4. The entire gel images of Figure 4.

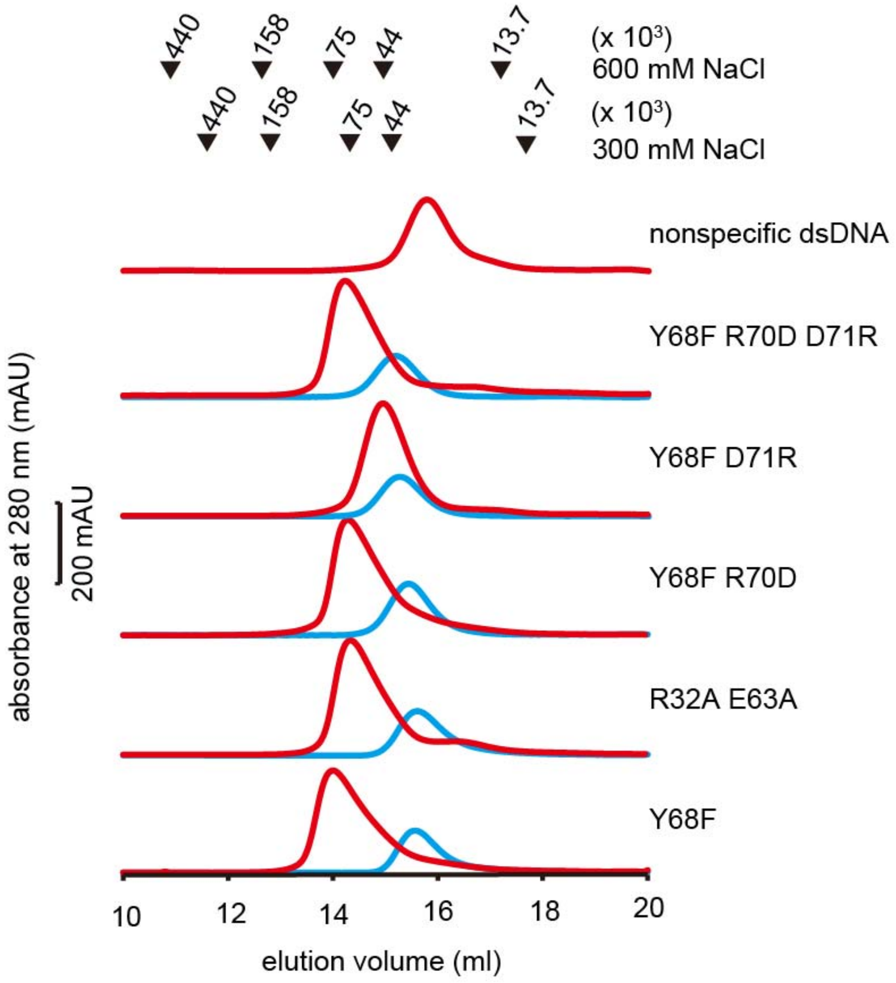

Supplementary Figure 5. Gel filtration analysis of the R.PabI mutants (cyan) and their nonspecific dsDNA complexes (red). The R.PabI Y68F D71R and Y68F R70D D71R mutants were eluted with buffer containing 10 mM MES (pH 6.0) and 600 mM NaCl to avoid nonspecific binding to the column. The other R.PabI mutants, the R.PabI-nonspecific dsDNA complexes, and the nonspecific dsDNA were eluted with buffer containing 10 mM MES (pH 6.0) and 300 mM NaCl. The peak positions of the marker proteins are indicated by the black triangles at the top of the chromatogram. The molecular weight of DNA free R.PabI Y68F, R32A E63A, Y68F R70D, Y68F D71R, and Y68F R70D D71R mutants are estimated to be 38,000, 38,000, 42,000, 38,000, and 40,000, respectively, indicating that R.PabI mutants form homodimers in solution (the molecular weight of R.PabI Y68F protomer is 25344). The molecular weight of R.PabI Y68F, R32A E63A, Y68F R70D, Y68F D71R, and Y68F R70D D71R mutants in complex with nonspecific dsDNA are estimated to be 86,000, 75,000, 78,000, 65,000, and 80,000, respectively. The R.PabI Y68F forms a homotetramer with nonspecific dsDNA (the molecular weight of nonspecific dsDNA is 12234). However, the tetramerization is prevented by the R70D and the D71R mutations.

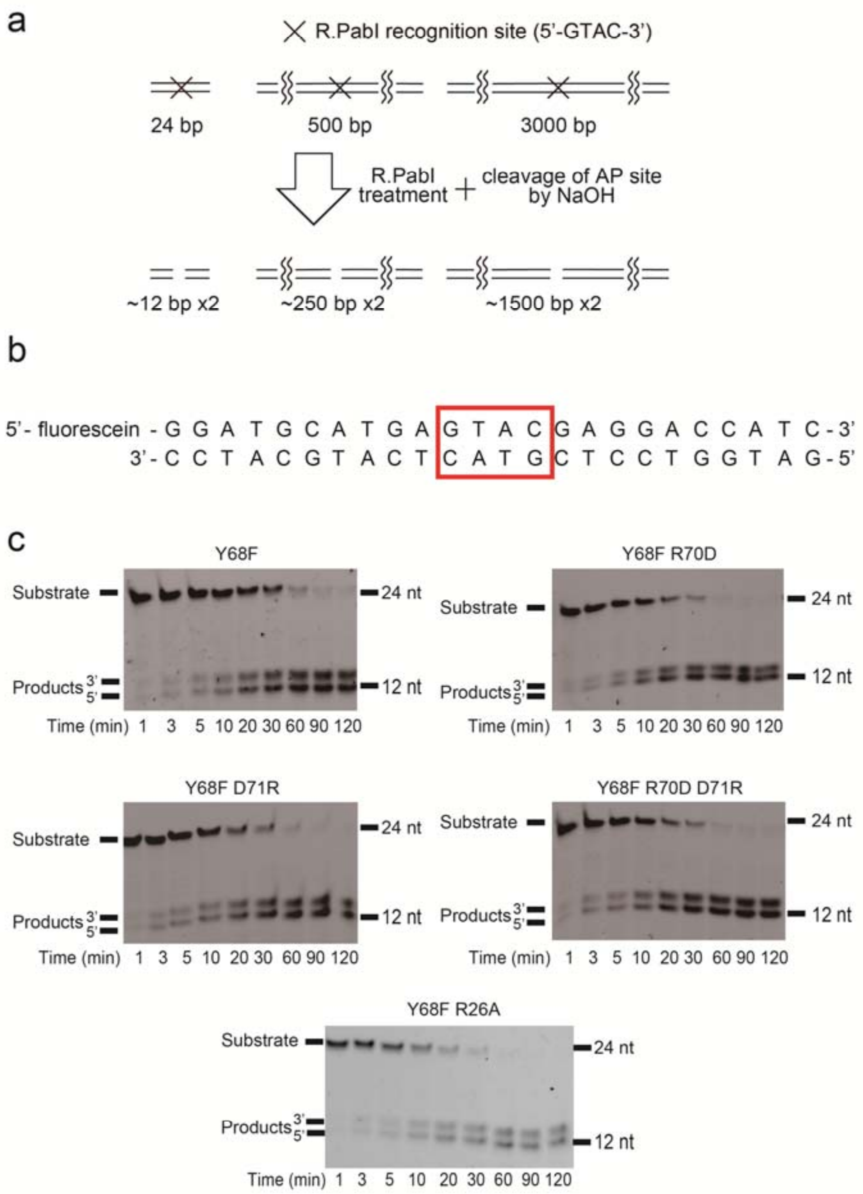
a
R.PabI recognition site (5'-GTAC-3')
24 bp
500 bp
3000 bp
R.PabI treatment
cleavage of AP site by NaOH
~12 bp x2
~250 bp x2
~1500 bp x2
b
5'- fluorescein - G G A T G C A T G A G T A C G A G G A C C A T C - 3'
3' - C C T A C G T A C T C A T G C T C C T G G T A G - 5'
c
Y68F
Y68F R70D
Y68F D71R
Y68F R70D D71R
Y68F R26A
Substrate
Products
3'
5'
24 nt
12 nt
Time (min) 1 3 5 10 20 30 60 90 120

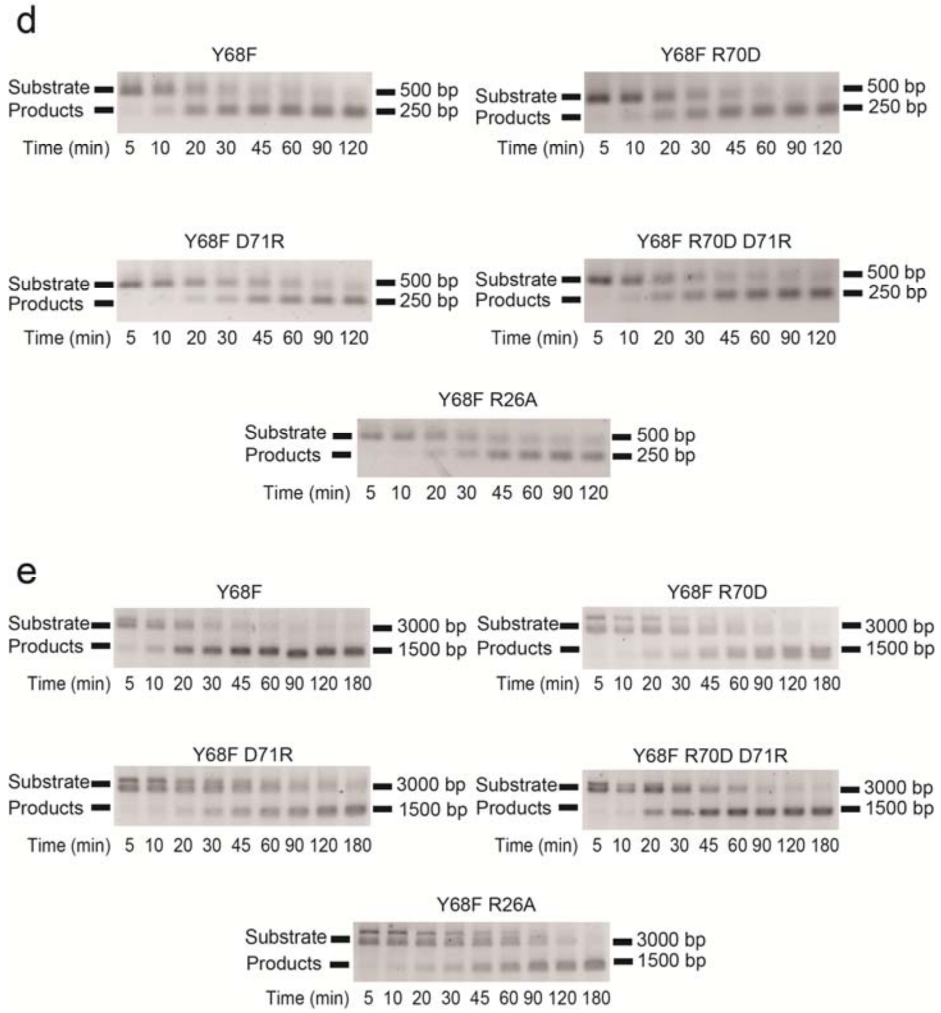

Supplementary Figure 6. DNA glycosylase activities of the R.PabI mutants. (**a**) Schematic diagram of three R.PabI substrates (24 bp, 500 bp, and 3000 bp) used in the DNA glycosylase assays. The recognition sequence (5'-GTAC-3') is located at the centre of each substrate. After R.PabI treatment and the cleavage of AP sites by NaOH, these substrates are separated into two products with equal lengths. (**b**) Sequence of dsDNA (24 bp) used for the DNA glycosylase assay. The R.PabI recognition sequence is indicated by a red box. (**c**) DNA glycosylase activity assay using 24 bp dsDNA as a substrate. First, 0.2 μM of the 24 bp substrate dsDNA and 0.4 μM of the R.PabI dimer were mixed and incubated at 45°C. Each experiment was repeated three times. (**d**) DNA glycosylase activity assay using 500 bp dsDNA as a substrate. First, 5.9 nM of the substrate DNA and 80 nM of the R.PabI dimer were mixed and incubated at 45°C. Each experiment was repeated three times. (**e**) DNA glycosylase activity assay using 3000 bp dsDNA as a substrate. First, 5.9 nM of the substrate DNA and 80 nM of the R.PabI dimer were mixed and incubated at 45°C. Each experiment was repeated three times.

Supplementary Table 1. Hydrogen bonds between R.PabI and DNA in the R.PabI-nonspecific dsDNA complex.

Protein				H-bonds	DNA			
Chain ID	Residue	Main/ Side	Atom Name	Distance (Å)	Chain ID	Base	Base/ Backbone	Atom Name
A	Thr46	Side	OG1	2.44	C’	Cyt4’	Backbone	OP2
A	Arg47	Main	N	2.96	C’	Cyt4’	Backbone	OP2
A	Arg47	Main	N	3.17	C’	Cyt4’	Backbone	O5’
A	Lys48	Main	N	3.41	C’	Cyt4’	Backbone	OP2
A	Lys48	Side	NZ	2.87	C’	Ade3’	Backbone	OP1
A	Lys49	Main	N	2.68	C’	Ade3’	Backbone	OP2
A	Ser50	Main	N	3.73	C’	Ade3’	Backbone	OP1
A	Ser50	Main	N	3.38	C’	Ade3’	Backbone	OP2
A	Ser29	Main	N	2.98	C	Ade12	Backbone	OP2
A	Lys30	Main	N	3.83	C	Ade12	Backbone	OP1
B	Thr25	Side	OG1	3.62	C’	Ade16’	Backbone	O3’
B	Thr25	Side	OG1	2.51	C’	Gua17’	Backbone	OP1
B	Ser45	Side	OG	3.70	C’	Ade16’	Backbone	OP1
B	Arg47	Side	NE	2.82	C’	Gua17’	Backbone	OP2
B	Arg156	Side	NH1	3.68	C’	Thy8’	Base	O4
B	Arg156	Side	NH1	2.70	C’	Gua7’	Base	O6
B	Val158	Main	N	3.38	C’	Thy5’	Backbone	OP1
B	Val158	Main	N	3.29	C’	Thy5’	Backbone	OP2
B	Arg184	Side	NE	3.24	C’	Thy18’	Backbone	OP2
B	Asn185	Side	N	2.76	C’	Gua17’	Backbone	OP1
B	Asn185	Side	ND2	3.17	C’	Gua17’	Backbone	O3’
B	Asn185	Side	ND2	3.33	C’	Thy18’	Backbone	OP1
B	Ser29	Main	N	2.76	C	Thy9	Backbone	OP1
B	Ser29	Side	OG	2.76	C	Thy9	Backbone	OP1
